# Risk of atrial fibrillation in persons with type 2 diabetes and the excess risk in relation to glycaemic control and renal function: a Swedish cohort study

**DOI:** 10.1186/s12933-019-0983-1

**Published:** 2020-01-18

**Authors:** Shilan Seyed Ahmadi, Ann-Marie Svensson, Aldina Pivodic, Annika Rosengren, Marcus Lind

**Affiliations:** 1 Department of Molecular and Clinical Medicine, University of Gothenburg, Gothenburg, Sweden; 2 Department of Medicine, NU-Hospital Group, Trollhättan/Uddevalla, Sweden; 3 Swedish National Diabetes Register, Centre of Registers, Gothenburg, Sweden; 4 Statistiska Konsultgruppen, Gothenburg, Sweden; 5 Department of Ophthalmology, Institute of Neuroscience and Physiology, Sahlgrenska Academy, University of Gothenburg, Gothenburg, Sweden; 6 Sahlgrenska University Hospital, Gothenburg, Sweden

**Keywords:** Albuminuria, Atrial fibrillation, Glycaemic control, Type 2 diabetes

## Abstract

**Background:**

To examine the incidence of atrial fibrillation in individuals with type 2 diabetes compared with age- and sex-matched controls from the general population and its variation in relation to glycaemic control and renal function.

**Methods:**

A total of 421,855 patients with type 2 diabetes from the Swedish National Diabetes Registry and 2,131,223 controls from the Swedish Population Registry, matched for age, sex and county, were included and followed from January 1, 2001 to December 31, 2013.

**Results:**

Overall, 8.9% of individuals with type 2 diabetes and 7.0% of controls were diagnosed with atrial fibrillation during follow-up, unadjusted incidence risk ratio (IRR) 1.35 (95% 1.33–1.36). Women < 55 years old with type 2 diabetes had an IRR of 2.36 (95% CI 2.10–2.66), in relation to controls, whereas the corresponding value for men < 55 years old with type 2 diabetes was IRR 1.78 (95% CI 1.67–1.90). In the fully adjusted Cox regression, the risk of type 2 diabetes on incident atrial fibrillation was 28% greater vs controls, hazard ratio (HR) 1.28 (95% CI 1.26–1.30), p < 0.0001. The excess risk of atrial fibrillation in individuals with type 2 diabetes increased with worsening glycaemic control and renal complications. For individuals with HbA1c ≤ 6.9% (≤ 52 mmol/mol) and normoalbuminuria the excess risk vs controls was still increased, adjusted HR 1.16 (95% CI 1.14–1.19); p < 0.0001.

**Conclusions:**

Individuals with type 2 diabetes had an overall 35% higher risk of atrial fibrillation compared to age- and sex-matched controls from the general population. The excess risk for atrial fibrillation increased with renal complications or with poor glycaemic control. Individuals with type 2 diabetes with good glycaemic control and normoalbuminuria had slightly increased risk.

## Background

Type 2 diabetes is a metabolic disorder, with an increased risk of micro- and macrovascular complications such as thromboembolism, stroke and mortality [1]. At the same time, atrial fibrillation is the most common cardiac arrhythmia with similar outcomes as type 2 diabetes [2]. The prevalence of both atrial fibrillation and type 2 diabetes has been rising rapidly [2, 3]. Studies have shown that when both conditions coexist, the risk for thromboembolism is increased [4].

There are conflicting findings on whether type 2 diabetes is an independent risk factor for atrial fibrillation [5–7]. However, several studies have shown that type 2 diabetes is associated with atrial fibrillation, with risk increasing with poor glycaemic control, glycaemic variability, longer diabetes duration [8], hypertension, and albuminuria [9]. In a meta-analysis, a dose-response relationship was found between increasing blood glucose and risk of atrial fibrillation [10]. Although extensive research has been carried out on the association between type 2 diabetes and atrial fibrillation, few subgroup analyses have been performed on glycaemic control and renal function, with none comparing the risk of atrial fibrillation in subgroups of patients with type 2 diabetes to age- and sex-matched controls from the general population.

Additionally, diabetes is one of the factors included in the risk stratification scheme CHA_2_DS_2_-VAS_c_-score used to identify high-risk patients with atrial fibrillation that may benefit from anticoagulation treatment in order to prevent stroke from thromboembolism [11]. However, guidelines are inadequate regarding recommendations for anticoagulation when both conditions coexist [4]. Hence, increased understanding is needed on how the risk of atrial fibrillation interacts with type 2 diabetes and coexisting diabetes-specific risk factors.

The Swedish National Diabetes Registry (NDR) provides nationwide information on patients with type 2 diabetes. The large size and widespread geographic distribution of the NDR provides unique opportunities to estimate excess risk for atrial fibrillation from both national and international perspectives. The purpose of this study was to examine (a) the incidence of atrial fibrillation in persons with type 2 diabetes and (b) how risk varies in relation to glycaemic control and renal function compared with age- and sex-matched controls from the general population.

## Methods

Ethical approval for this study was obtained from the Regional Ethical Review Board in Gothenburg, Sweden.

### Data sources

We conducted a registry-based cohort study, obtaining data from registries that have nationwide coverage. Clinical data in the NDR are reported from clinical visits in primary care and hospital outpatient clinics regarding risk factors, diabetes-related complications, and medications for individuals with diabetes (type 1 and 2) ≥ 18 years old. Each patient provides informed consent for inclusion in the NDR.

The National Patient Registry (NPR) was used to obtain information on atrial fibrillation, outcomes and co-morbidities and contains records on all admissions to hospitals and outpatient specialist visits, including data such as date of contact, diagnostic, and procedure codes. The population registries and the central bureaus of statistics provided demographic, vital status, and socioeconomic data.

Information about mortality was retrieved from the Swedish Cause of Death Registry. Information about educational level and country of birth was accessed from the Longitudinal Integration Database for Health Insurance and Labor Market Studies. Education level was categorized as low (compulsory education), intermediate, or high (university level education or similar) and country of birth as Sweden or other.

### Study population

Criteria for inclusion in the study were individuals with type 2 diabetes with at least one registration in the NDR. Type 2 diabetes was defined as treatment with either diet or oral antihyperglycemic agents only or diagnosis at ≥ 40 years of age receiving insulin therapy or insulin and oral antihyperglycemic agents. In total 421,855 individuals with type 2 diabetes were included in the study. For each person, five controls without type 2 diabetes were selected randomly from the Swedish Population Registry matched for age, sex, and county. In total, 2,131,223 controls were selected.

Individuals with type 2 diabetes who died [2688 (0.10%)], had atrial fibrillation before January 1, 2001 or before index date [41,547 (1.52%)] or no NDR visits after January 1, 2001 [7712 (0.28%)] were excluded (data entry prior to January 1, 1999 was incomplete when comparing care units). Controls who died before January 1, 2001 [11,427 (0.42%)] or had atrial fibrillation before January 1, 2001 or before index date [121,142 (4.43%)] were also excluded.

### Study procedures

Variables assessed were sex, age, country of birth, educational level, HbA1c (glycated haemoglobin), diabetes duration, BMI (body weight in kilograms divided by height in m^2^), low-density lipoprotein (LDL) cholesterol, systolic and diastolic blood pressure, smoking, renal function (eGFR), and albuminuria.

The incidence of atrial fibrillation was calculated stratified by sex, age group, glycaemic control, and renal function. Glycaemic control was estimated by updated mean HbA1c, which is the mean value of HbA1c measurements up until a certain point in time. Patients were categorized by glycaemic control based on the mean level of HbA1c until a certain time point. HbA1c was measured according to the International Federation of Clinical Chemistry (IFCC) method in mmol/mol and converted to levels according to the National Glycohemoglobin Standardization Program for dual reporting.

Renal complications were categorized as normoalbuminuria, microalbuminuria, macroalbuminuria, and stage 1–5 chronic kidney disease (CKD). Microalbuminuria was defined as two positive tests from three urine samples taken within 1 year, with an albumin/creatinine ratio of 3–30 mg/mmol (~ 30–300 mg/g) or U-albumin of 20–200 μg/min (20–300 mg/L), and macroalbuminuria as albumin/creatinine ratio > 30 mg/mmol (~>300 mg/g) or U-albumin > 200 μg/min (> 300 mg/L). Estimated glomerular filtration rate (eGFR) was calculated using the Chronic Kidney Disease Epidemiology (CKD-EPI) equation [12]. Renal function was categorized as CKD stage 1 (eGFR ≥ 90 mL/min), stage 2 (eGFR 60–89 mL/min), stage 3 (eGFR 30–59 mL/min), stage 4 (eGFR 15–29 mL/min) and CKD stage 5 (eGFR < 15 mL/min or need for renal dialysis).

### Study outcome and comorbidities

The study outcome is atrial fibrillation and flutter, the ICD codes 427D (ICD-9) and I48 (ICD-10), with both primary and secondary diagnosis. International Classification of Disease 9th (ICD-9) and 10th (ICD-10) codes were used to define study comorbidities coronary heart disease, admission for heart failure, valve disease, stroke, and cancer diagnosis before the first and baseline registration (Additional file 1).

### Statistical analysis

Crude event rates were described in total, by age category (< 55, 55–64, 65–74 and > 75 years), and by gender as events per 1000 person-years with 95% confidence intervals estimated using Poisson regression. The effects of time-updated mean HbA1c categories (mean of all preceding values at each timepoint), time-updated albuminuria categories (last of all preceding values at each timepoint), and time-updated eGFR categories (last of all preceding values at each timepoint) versus the reference group on time to incident atrial fibrillation were analyzed using Cox regression in three different models. In model 1, adjustments were made for time-updated age and sex. In model 2, adjustments were made for country of birth, education, and baseline comorbidities (coronary heart disease, heart failure, valve disease, stroke and cancer). In model 3, additional adjustments were made for time-updated diabetes duration. The effects of diabetes group overall and of HbA1c, albuminuria and eGFR categories versus the normal population were obtained for 10th, 25th, 50th, 75th, and 90th percentile of diabetes duration, corresponding to 1, 3, 7, 13 and 19 years. Main analyses were shown for median diabetes duration of 7 years.

In individuals with diabetes, overall and for women and men separately, the association of a 1.0% (10 mmol/mol) increase in time-updated mean HbA1c was evaluated with incidence of atrial fibrillation. Adjustments in models 1, 2, and 3 correspond to the main analyses adjustments. Further adjustments were done in models A, B, C, and D. Model A, adjustment was done for time-updated mean systolic blood pressure, time-updated mean body mass index, time updated smoking status and time-updated status about blood pressure lowering medication. In model B, adjustments for time-updated mean high-density lipoprotein, time-updated mean low-density lipoprotein, time-updated status about lipid lowering medication were done. Model C were additionally adjusted for time-updated insulin method. In model D, adjustments for time-updated albuminuria categories were done. These analyses have been described earlier [13]. The proportional hazards assumption was checked through visual review of log (– log [survival]) versus log(time) curves.

All tests were two-tailed with p-values < 0.05 considered significant. All analyses were performed using SAS software version 9.4 (SAS Institute Inc., Cary, NC, USA).

## Results

### Study population

A total of 421,855 patients with type 2 diabetes and 2,131,223 controls were included. Patient characteristics at baseline are presented in Table 1. Among people with type 2 diabetes, mean age was 64.7 ± 12.5 years vs 64.6 ± 12.5 years for controls. The mean HbA1c was 7.1 ± 1.4 % (54.3 ± 14.9 mmol/mol) with a mean duration of diabetes of 5.4 ± 7.1 years. Individuals with high HbA1c values were younger, had a higher mean LDL-level, more frequently smokers, and were more frequently born outside of Sweden.

**Table 1:** Baseline characteristics

	Controls n = 2,131,223	All Type 2 diabetes n = 421,855	HbA1c categories at baseline (NGSP %/IFCC mmol/mol)					
			≤ 6.9% (≤52 mmol/mol) n = 210,655	7.0–7.8% (53–62 mmol/mol) n = 79,876	7.9–8.7% (63–72 mmol/mol) n = 42,230	8.8–9.6% (73–82 mmol/mol) n = 20,169	≥ 9.7% (≥83 mmol/mol) n = 19,842	Missing value n = 49,083
Women	975,940 (45.8%)	188,307 (44.6%)	96,826 (46.0%)	35,228 (44.1%)	18,222 (43.1%)	8287 (41.1%)	7590 (38.3%)	22,154 (45.1%)
Age (years)	64.6 (12.5) n = 2,131,223	64.7 (12.5) n = 421,855	65.4 (12.2) n = 210,655	65.9 (12.0) n = 79,876	64.6 (12.3) n = 42,230	62.8 (12.5) n = 20,169	60.2 (12.6) n = 19,842	62.7 (14.0) n = 49,083
Age category								
< 55 years	424,662 (19.9%)	83,356 (19.8%)	37,566 (17.8%)	13,480 (16.9%)	8490 (20.1%)	4983 (24.7%)	6261 (31.6%)	12,576 (25.6%)
55–64 years	606,294 (28.4%)	118,424 (28.1%)	57,891 (27.5%)	22,234 (27.8%)	12,254 (29.0%)	6170 (30.6%)	6501 (32.8%)	13,374 (27.2%)
65–74 years	623,184 (29.2%)	122,848 (29.1%)	64,271 (30.5%)	23,789 (29.8%)	11,855 (28.1%)	5252 (26.0%)	4459 (22.5%)	13,222 (26.9%)
75 + years	477,083 (22.4%)	97,227 (23.0%)	50,927 (24.2%)	20,373 (25.5%)	9631 (22.8%)	3764 (18.7%)	2621 (13.2%)	9911 (20.2%)
Born in Sweden	1,862,552 (87.4%)	343,748 (81.6%)	175,599 (83.4%)	65,386 (81.9%)	34,031 (80.7%)	16,015 (79.5%)	15,134 (76.4%)	37,583 (76.6%)
Education category								
Low	751,922 (35.9%)	176,826 (42.9%)	87,269 (42.3%)	35,461 (45.5%)	18,982 (46.2%)	8804 (45.0%)	7772 (40.4%)	18,538 (39.0%)
Mid	833,045 (39.8%)	168,052 (40.8%)	84,216 (40.8%)	31,074 (39.9%)	16,334 (39.8%)	7942 (40.6%)	8488 (44.1%)	19,998 (42.0%)
High	510,647 (24.4%)	66,930 (16.3%)	35,003 (17.0%)	11,322 (14.5%)	5758 (14.0%)	2797 (14.3%)	2993 (15.5%)	9057 (19.0%)
Variables in the NDR only								
HbA1c (mmol/mol, %)		54.3 (14.9) 7.12 (1.36) n = 372,772	44.7 (5.0) 6.25 (0.46) n = 210,655	56.5 (2.6) 7.33 (0.24) n = 79,876	66.9 (2.8) 8.27 (0.26) n = 42,230	76.9 (2.8) 9.19 (0.26) n = 20,169	97.2 (12.6) 11.04 (1.16) n = 19,842	
Diabetes duration (years)		5.35 (7.08) n = 384,096	3.98 (5.72) n = 192,553	6.61 (7.25) n = 74,795	8.01 (7.91) n = 39,781	7.94 (8.05) n = 19,041	5.46 (7.31) n = 18,556	5.61 (9.29) n = 39,370
BMI (kg/m2)		29.8 (5.4) n = 316,107	29.5 (5.2) n = 172,985	30.0 (5.4) n = 66,304	30.1 (5.4) n = 34,836	30.5 (5.7) n = 16,412	30.5 (5.9) n = 15,503	30.1 (5.7) n = 10,067
LDL (mmol/L, mg/dL)		2.96 (0.97) 114.4 (37.3) n = 224,597	2.94 (0.94) 113.6 (36.5) n = 128,210	2.94 (0.96) 113.5 (37.2) n = 45,692	2.94 (0.98) 113.6 (37.7) n = 22,361	3.00 (1.00) 115.7 (38.5) n = 10,195	3.25 (1.09) 125.5 (42.0) n = 9864	3.09 (1.00) 119.2 (38.8) n = 8275
Systolic Blood Pressure (mmHg)		140.0 (18.0) n = 359,010	139.0 (17.6) n = 196,021	141.2 (18.1) n = 74,411	141.7 (18.3) n = 38,960	141.9 (18.7) n = 18,305	140.5 (19.0) n = 17,111	139.1 (18.9) n = 14,202
Distolic Blood Pressure (mmHg)		78.7 (9.8) n = 359,010	78.2 (9.6) n = 196,021	78.7 (9.8) n = 74,411	79.2 (9.9) n = 38,960	80.0 (10.0) n = 18,305	81.4 (10.4) n = 17,111	79.3 (10.6) n = 14,202
Smoker								
No		279,514 (83.7%)	153,404 (85.3%)	58,293 (84.8%)	30,196 (83.6%)	13,833 (81.2%)	12,477 (78.7%)	11,311 (69.9%)
Yes		54,265 (16.3%)	26,412 (14.7%)	10,477 (15.2%)	5925 (16.4%)	3196 (18.8%)	3386 (21.3%)	4869 (30.1%)
eGFR (MDRD) (cat.)								
CKD stage 1 (eGFR ≥90)		96,846 (32.2%)	48,373 (29.2%)	18,970 (31.3%)	10,514 (34.9%)	5758 (40.5%)	7391 (51.2%)	5840 (35.9%)
CKD stage 2 (eGFR 60–89)		153,262 (50.9%)	89,151 (53.9%)	30,699 (50.7%)	14,332 (47.5%)	6132 (43.2%)	5356 (37.1%)	7592 (46.7%)
CKD stage 3 (eGFR 30–59)		47,880 (15.9%)	26,378 (15.9%)	10,258 (16.9%)	4954 (16.4%)	2153 (15.2%)	1579 (10.9%)	2558 (15.7%)
CKD stage 4 (eGFR 15–29)		2491 (0.8%)	1208 (0.7%)	537 (0.9%)	293 (1.0%)	142 (1.0%)	102 (0.7%)	209 (1.3%)
CKD stage 5 (eGFR < 15 or dialysis)		508 (0.2%)	277 (0.2%)	66 (0.1%)	54 (0.2%)	18 (0.1%)	19 (0.1%)	74 (0.5%)
eGFR (CKD-EPI) (cat.)								
CKD stage 1 (eGFR ≥ 90)		112,140 (37.3%)	57,044 (34.5%)	21,754 (35.9%)	12,012 (39.8%)	6583 (46.3%)	8275 (57.3%)	6472 (39.8%)
CKD stage 2 (eGFR 60–89)		142,152 (47.2%)	82,943 (50.2%)	28,705 (47.4%)	13,194 (43.8%)	5529 (38.9%)	4698 (32.5%)	7083 (43.5%)
CKD stage 3 (eGFR 30–59)		43,302 (14.4%)	23,707 (14.3%)	9374 (15.5%)	4558 (15.1%)	1910 (13.4%)	1341 (9.3%)	2412 (14.8%)
CKD stage 4 (eGFR 15–29)		2850 (0.9%)	1396 (0.8%)	626 (1.0%)	325 (1.1%)	160 (1.1%)	114 (0.8%)	229 (1.4%)
CKD stage 5 (eGFR < 15 or dialysis)		543 (0.2%)	297 (0.2%)	71 (0.1%)	58 (0.2%)	21 (0.1%)	19 (0.1%)	77 (0.5%)
Registrations in the IPR prior to baseline								
Coronary heart disease (I20–I25)	137,684 (6.5%)	59,008 (14.0%)	29,142 (13.8%)	12,099 (15.1%)	6360 (15.1%)	2895 (14.4%)	2224 (11.2%)	6288 (12.8%)
Heart failure (I50)	36,679 (1.7%)	16,996 (4.0%)	7578 (3.6%)	3512 (4.4%)	2026 (4.8%)	1041 (5.2%)	848 (4.3%)	1991 (4.1%)
Valve disease (I05–I09, I34–I36)	23,120 (1.1%)	6079 (1.4%)	3279 (1.6%)	1133 (1.4%)	539 (1.3%)	264 (1.3%)	186 (0.9%)	678 (1.4%)
Stroke (I61–I64)	64,724 (3.0%)	22,417 (5.3%)	11,318 (5.4%)	4317 (5.4%)	2380 (5.6%)	1007 (5.0%)	802 (4.0%)	2593 (5.3%)
Cancer (C00–C97)	176,394 (8.3%)	37,135 (8.8%)	19,885 (9.4%)	6944 (8.7%)	3198 (7.6%)	1421 (7.0%)	1245 (6.3%)	4442 (9.0%)

For categorical variables n (%) is presented. For continuous variables Mean (SD) is presented

### Excess risk of atrial fibrillation in relation to age and sex

The mean duration of follow-up was 5.0 years for individuals with type 2 diabetes and 5.3 years for controls. Between January 1, 2001, and December 31, 2013, a total of 37,590 (8.9%) individuals with type 2 diabetes and 149,231 (7.0%) of controls were diagnosed with atrial fibrillation. Overall, for individuals with type 2 diabetes, the incidence rates of atrial fibrillation were 15.99 cases per 1000 person-years (95% CI 15.83–16.15) compared to 11.89 cases per 1000 person-years (95% CI 11.83–11.95) for the control group (Table 2), unadjusted incidence risk ratio (IRR) 1.35 (95% CI 1.33–1.36). As seen in Table 2, when stratified by age, the incidences rates of atrial fibrillation increased with age for both individuals with type 2 diabetes and controls; however, the relative risk between the two groups decreased with older age.

**Table 2:** Atrial fibrillation per 1000 patient years by sex and age categories at baseline with 95% confidence intervals estimated by using Poisson regression

Atrial fibrillation, any position	All		Men		Women	
	Type 2 diabetes	Controls	Type 2 diabetes	Controls	Type 2 diabetes	Controls
All						
N	421,855	2,131,223	233,548	1,155,283	188,307	975,940
n (%)	37,590 (8.9%)	149,231 (7.0%)	21,808 (9.3%)	86,178 (7.5%)	15,782 (8.4%)	63,053 (6.5%)
Cases per 1000 years (95% CI)	15.99 (15.83–16.15)	11.89 (11.83–11.95)	16.95 (16.73–17.18)	12.66 (12.58–12.75)	14.83 (14.60–15.07)	10.97 (10.88–11.05)
Unadjusted IRR (95% CI)	1.35 (1.33–1.36)		1.34 (1.32–1.36)		1.35 (1.33–1.38)	
< 55 years						
N	83,356	424,662	50,094	255,240	33,262	169,422
n (%)	1569 (1.9%)	4356 (1.0%)	1175 (2.3%)	3478 (1.4%)	394 (1.2%)	878 (0.5%)
Cases per 1000 years (95% CI)	3.18 (3.02–3.34)	1.67 (1.62–1.72)	3.96 (3.74–4.20)	2.23 (2.15–2.30)	1.99 (1.80–2.20)	0.84 (0.79–0.90)
Unadjusted IRR (95% CI)	1.90 (1.79–2.01)		1.78 (1.67–1.90)		2.36 (2.10–2.66)	
55–64 years						
N	118,424	606,294	72,337	371,480	46,087	234,814
n (%)	6819 (5.8%)	22,332 (3.7%)	4901 (6.8%)	16,729 (4.5%)	1918 (4.2%)	5603 (2.4%)
Cases per 1000 years (95% CI)	9.50 (9.28–9.73)	5.84 (5.77–5.92)	11.30 (10.99–11.62)	7.20 (7.09–7.31)	6.75 (6.46–7.06)	3.74 (3.64–3.84)
Unadjusted IRR (95% CI)	1.63 (1.58–1.67)		1.57 (1.52–1.62)		1.81 (1.71–1.90)	
65–74 years						
N	122,848	623,184	67,666	335,590	55,182	287,594
n (%)	12,973 (10.6%)	50,919 (8.2%)	8011 (11.8%)	32,223 (9.6%)	4962 (9.0%)	18,696 (6.5%)
Cases per 1000 years (95% CI)	18.99 (18.67–19.32)	13.75 (13.63–13.87)	22.10 (21.62–22.59)	16.54 (16.36–16.72)	15.47 (15.05–15.91)	10.65 (10.50–10.80)
Unadjusted IRR (95% CI)	1.38 (1.36–1.41)		1.34 (1.30–1.37)		1.45 (1.41–1.50)	
75 + years						
N	97,227	477,083	43,451	192,973	53,776	284,110
n (%)	16,229 (16.7%)	71,624 (15.0%)	7721 (17.8%)	33,748 (17.5%)	8508 (15.8%)	37,876 (13.3%)
Cases per 1000 years (95% CI)	35.62 (35.08–36.17)	29.54 (29.33–29.76)	39.76 38.88–40.66)	34.82 (34.45–35.20)	32.54 (31.86–33.24)	26.02 (25.76–26.29)
Unadjusted IRR (95% CI)	1.21 (1.19–1.23)		1.14 (1.11–1.17)		1.25 (1.22–1.28)	

In Poisson regression analyses, the IRRs for atrial fibrillation in men and women with type 2 diabetes compared to controls were similar, 1.34 vs 1.35, respectively (Table 2). When stratified by age, the absolute risk for atrial fibrillation was higher for men with type 2 diabetes, but the relative risk was greater for women with type 2 diabetes compared to their matched controls. For men, incidence rates of atrial fibrillation for patients with type 2 diabetes were 16.95 cases per 1000 person-years (95% CI 16.73–17.18) and for controls 12.66 cases per 1000 person-years (95% CI 12.58–12.75). For women, the corresponding values were 14.83 cases per 1000 years (95% CI 14.60–15.07) vs controls 10.97 cases per 1000 person-years (95% CI 10.88–11.05). The unadjusted IRRs for men with type 2 diabetes aged < 55 years old were 1.78 (95% CI 1.67–1.90) and for men > 75 years old the IRR was reduced to 1.14 (95% CI 1.11–1.17) compared to controls. For women, the corresponding estimates were 2.36 (95% CI 2.10–2.66) and 1.25 (95% CI 1.22–1.28), respectively, compared to controls.

The excess risk for atrial fibrillation was evaluated in relation to diabetes duration, and effects were evaluated for median diabetes duration of 7 years (Additional file 1: Table S1). In model 1, the incidences for atrial fibrillation were adjusted for time-updated age and sex. For individuals with type 2 diabetes, the HR was 1.37 (95% CI 1.35–1.38), p < 0.0001 compared to controls. When further adjustment for country of birth, education and baseline comorbidities was performed (model 2), the HR decreased to 1.30 (95% CI 1.28–1.31), p < 0.0001 compared to controls. In model 3, with additional adjustments for time-updated diabetes duration, the HR decreased to 1.28 (95% CI 1.26–1.30), p < 0.0001 compared to controls. Corresponding estimates of risk for atrial fibrillation stratified by sex are shown in Additional file 1: Table S1. In model 3, women with type 2 diabetes had an HR of 1.31 (95% CI 1.28–1.34) compared to controls, whereas the corresponding value for men with type 2 diabetes was 1.26 (95% 1.24–1.28). For individuals with type 2 diabetes, the HR per 10-year increase for time-updated diabetes duration was 1.03 (95% CI 1.02–1.05); p < 0.0001 for all, 1.04 (95% CI 1.02–1.06); p < 0.0001 for men and for women 1.02 (95% CI 1.00–1.05); p = 0.068.

### Excess risk for atrial fibrillation for time-updated mean HbA1c, albuminuria, and eGFR categories

Table 3 displays the risk of atrial fibrillation by categories of time-updated HbA1c, albuminuria, and eGFR adjusted according to model 3 in individuals with type 2 diabetes versus controls. There was a significant excess risk with increasing HbA1c and albuminuria. The increased risk was also present for individuals with type 2 diabetes and good glycaemic control or normoalbuminuria, with HRs of 1.24 (95% CI 1.22–1.26), p < 0.0001, and 1.17 (95% CI 1.16–1.19), p < 0.0001, compared to controls, respectively. The corresponding values from models 1 and 2 and for men and women, respectively, are found in Additional file 1: Table S2 and S3.

**Table 3:** Adjusted hazard ratios for atrial fibrillation and 95% confidence intervals for time-updated mean HbA1c categories, albuminuria categories and eGFR categories versus the reference group examined by Cox regression

Atrial fibrillation	Hazard ratio (95% CI) *p* value		
	Model 1	Model 2	Model 3
Time updated mean HbA1c categories	n events = 185,793 N subjects = 2,544,103 data used = 99.6%	n events = 182,081 N subjects = 2,498,727 data used = 97.9%	n events = 179,445 N subjects = 2,465,540 data used = 96.6%
Controls (reference)	1.00	1.00	1.00
≤ 6.9% (≤ 52 mmol/mol)	1.32 (1.30–1.34) < 0.0001	1.26 (1.24–1.27) < 0.0001	1.24 (1.22–1.26) < 0.0001
7.0–7.8% (53–62 mmol/mol)	1.36 (1.34–1.39) < 0.0001	1.29 (1.27–1.32) < 0.0001	1.28 (1.25–1.31) < 0.0001
7.9–8.7% (63–72 mmol/mol)	1.47 (1.43–1.52) < 0.0001	1.38 (1.34–1.42) < 0.0001	1.38 (1.34–1.42) < 0.0001
8.8–9.6% (73–82 mmol/mol)	1.61 (1.54–1.69) < 0.0001	1.49 (1.42–1.57) < 0.0001	1.47 (1.40–1.55) < 0.0001
≥ 9.7% (≥ 83 mmol/mol)	1.65 (1.55–1.77) < 0.0001	1.56 (1.46–1.68) < 0.0001	1.57 (1.46–1.68) < 0.0001
Time updated albuminuria categories	n events = 179,380 N subjects = 2,473,810 Data used = 96.9%	n events = 175,890 N subjects = 2,431,009 Data used = 95.2%	n events = 174,059 N subjects = 2,409,479 Data used = 94.4%
Controls (reference)	1.00	1.00	1.00
Normoalbuminuria	1.24 (1.22–1.25) < 0.0001	1.18 (1.17–1.20) < 0.0001	1.17 (1.16–1.19) < 0.0001
Microalbuminuria	1.63 (1.59–1.67) < 0.0001	1.54 (1.50–1.58) < 0.0001	1.52 (1.48–1.56) < 0.0001
Macroalbuminuria	1.91 (1.85–1.97) < 0.0001	1.75 (1.69–1.80) < 0.0001	1.74 (1.68–1.80) < 0.0001
CKD stage 5	3.73 (3.32–4.20) < 0.0001	3.23 (2.87–3.64) < 0.0001	3.24 (2.86–3.66) < 0.0001
Time updated eGFR categories	n events = 183,107 N subjects = 2,525,557 Data used = 98.9%	n events = 179,482 N subjects = 2,481,018 Data used = 97.2%	n events = 176,958 N subjects = 2,449,089 Data used = 95.9%
Controls (reference)	1.00	1.00	1.00
CKD stage 1 (eGFR ≥ 90)	1.38 (1.34–1.42) < 0.0001	1.35 (1.31–1.39) < 0.0001	1.33 (1.29–1.37) < 0.0001
CKD stage 2 (eGFR 60–89)	1.28 (1.26–1.30) < 0.0001	1.23 (1.21–1.25) < 0.0001	1.22 (1.20–1.24) < 0.0001
CKD stage 3 (eGFR 30–59)	1.47 (1.44–1.50) < 0.0001	1.34 (1.32–1.37) < 0.0001	1.33 (1.30–1.36) < 0.0001
CKD stage 4 (eGFR 15–29)	2.09 (1.98–2.21) < 0.0001	1.78 (1.68–1.88) < 0.0001	1.76 (1.66–1.87) < 0.0001
CKD stage 5 (eGFR < 15 or dialysis)	3.77 (3.36–4.25) < 0.0001	3.27 (2.91–3.69) < 0.0001	3.24 (2.87–3.67) < 0.0001

Model 1: adjusted for time-updated age and sex

Model 2: model 1 additionally adjusted for born in Sweden, maximum education level and baseline comorbidities (CHD, HF, VD, stroke, cancer)

Model 3: model 2 additionally adjusted for time-updated diabetes duration (effects taken for DD = 7 years [median])

### Excess risk for atrial fibrillation related to time-updated mean HbA1c together with albuminuria and eGFR

In Table 4 the excess risk of atrial fibrillation was evaluated in relation to HbA1c for presence/absence of albuminuria versus controls. Individuals with HbA1c ≤ 6.9% (≤ 52 mmol/mol) and normoalbuminuria had an HR of 1.16 (95% CI 1.14–1.19) compared to controls. The corresponding value for individuals with HbA1c ≥ 9.7% (≥ 83 mmol/mol) and normoalbuminuria was 1.35 (95% CI 1.20–1.52). Corresponding HRs were 1.55 (95% 1.50–1.60) and 2.04 (95% CI 1.82–2.28) vs controls for individuals with HbA1c ≤ 6.9% (≤ 52 mmol/mol) and ≥ 9.7% (≥ 83 mmol/mol) with albuminuria, respectively. This was also evaluated in men and women separately (Additional file 1: Table S4 and S5). An increasing excess risk versus controls for men and women was noted with higher time-updated mean HbA1c for patients with normoalbuminuria, albuminuria as well as within different eGFR categories.

**Table 4:** Adjusted hazard ratios for atrial fibrillation and 95% confidence intervals for time-updated mean HbA1c categories together with albuminuria and eGFR versus the reference group examined by Cox regression

Atrial fibrillation	Hazard ratio (95% CI) p-value		
	Model 1	Model 2	Model 3
Time updated mean HbA1c categories and albuminuria	n events = 179,166 N subjects = 2,471,982 Data used = 96.8%	n events = 175,683 N subjects = 2,429,261 Data used = 95.2%	n events = 173,899 N subjects = 2,408,243 Data used = 94.3%
Controls (reference)	1.00	1.00	1.00
≤ 6.9% (≤ 52 mmol/mol)–normoalbuminuria	1.22 (1.20–1.24) < 0.0001	1.17 (1.15–1.20) < 0.0001	1.16 (1.14–1.19) < 0.0001
7.0–7.8% (53–62 mmol/mol)–normoalbuminuria	1.22 (1.19–1.25) < 0.0001	1.16 (1.13–1.20) < 0.0001	1.16 (1.13–1.19) < 0.0001
7.9–8.7% (63–72 mmol/mol)–normoalbuminuria	1.30 (1.24–1.35) < 0.0001	1.23 (1.17–1.28) < 0.0001	1.24 (1.18–1.29) < 0.0001
8.8–9.6% (73–82 mmol/mol)–Normoalbuminuria	1.40 (1.30–1.51) < 0.0001	1.32 (1.23–1.43) < 0.0001	1.31 (1.21–1.42) < 0.0001
≥ 9.7% (≥ 83 mmol/mol)–normoalbuminuria	1.43 (1.28–1.61) < 0.0001	1.35 (1.20–1.51) < 0.0001	1.35 (1.20–1.52) < 0.0001
≤ 6.9% (≤ 52 mmol/mol)–Not normoalbuminuria	1.68 (1.63–1.73) < 0.0001	1.56 (1.52–1.61) < 0.0001	1.55 (1.50–1.60) < 0.0001
7.0–7.8% (53–62 mmol/mol)–Not normoalbuminuria	1.72 (1.67–1.79) < 0.0001	1.62 (1.56–1.67) < 0.0001	1.61 (1.55–1.67) < 0.0001
7.9–8.7% (63–72 mmol/mol)–Not normoalbuminuria	1.86 (1.77–1.95) < 0.0001	1.72 (1.64–1.81) < 0.0001	1.73 (1.64–1.82) < 0.0001
8.8–9.6% (73–82 mmol/mol)–Not normoalbuminuria	2.04 (1.90–2.20) < 0.0001	1.85 (1.71–1.99) < 0.0001	1.85 (1.71–2.00) < 0.0001
≥ 9.7% (≥ 83 mmol/mol)–Not normoalbuminuria	2.20 (1.97–2.45) < 0.0001	2.00 (1.79–2.23) < 0.0001	2.04 (1.82–2.28) < 0.0001
Time updated mean HbA1c categories and eGFR	n events = 182,743 N subjects = 2,522,210 Data used = 98.8%	n events = 179,127 N subjects = 2,477,785 Data used = 97.1%	n events = 176,733 N subjects = 2,447,085 Data used = 95.8%
Controls (reference)	1.00	1.00	1.00
≤ 6.9% (≤ 52 mmol/mol) –eGFR ≥ 60	1.26 (1.24–1.29) < 0.0001	1.22 (1.20–1.25) < 0.0001	1.21 (1.19–1.24) < 0.0001
7.0–7.8% (53- 62 mmol/mol)–eGFR ≥ 60	1.29 (1.26–1.32) < 0.0001	1.24 (1.21–1.28) < 0.0001	1.24 (1.20–1.27) < 0.0001
7.9–8.7% (63–72 mmol/mol)–eGFR ≥ 60	1.38 (1.33–1.44) < 0.0001	1.33 (1.27–1.38) < 0.0001	1.33 (1.27–1.39) < 0.0001
8.8–9.6% (73–82 mmol/mol)–eGFR ≥ 60	1.51 (1.41–1.61) < 0.0001	1.43 (1.34–1.53) < 0.0001	1.41 (1.32–1.52) < 0.0001
≥ 9.7% (≥ 83 mmol/mol)–eGFR >=60	1.55 (1.42–1.71) < 0.0001	1.51 (1.38–1.66) < 0.0001	1.52 (1.38–1.68) < 0.0001
≤ 6.9% (≤ 52 mmol/mol)–eGFR < 60	1.45 (1.42–1.49) < 0.0001	1.33 (1.30–1.37) < 0.0001	1.32 (1.28–1.36) < 0.0001
7.0–7.8% (53–62 mmol/mol)–eGFR < 60	1.54 (1.49–1.60) < 0.0001	1.41 (1.36–1.46) < 0.0001	1.40 (1.35–1.45) < 0.0001
7.9–8.7% (63–72 mmol/mol)–eGFR < 60	1.73 (1.65–1.81) < 0.0001	1.55 (1.47–1.62) < 0.0001	1.56 (1.48–1.65) < 0.0001
8.8–9.6% (73–82 mmol/mol)–eGFR < 60	1.87 (1.72–2.03) < 0.0001	1.64 (1.51–1.79) < 0.0001	1.65 (1.51–1.80) < 0.0001
≥ 9.7% (≥ 83 mmol/mol)–eGFR < 60	1.98 (1.74–2.25) < 0.0001	1.71 (1.50–1.95) < 0.0001	1.75 (1.53–2.00) < 0.0001
Time updated mean HbA1c categories albuminuria and eGFR	n events = 179,980 N subjects = 2,480,217 Data used = 97.1%	n events = 176,459 N subjects = 2,437,158 Data used = 95.5%	n events = 174,466 N subjects = 2,414,092 Data used = 94.6%
Controls (reference)	1.00	1.00	1.00
≤ 6.9% (≤ 52 mmol/mol)–Normoalbuminuria and eGFR ≥60	1.19 (1.16–1.22) < 0.0001	1.16 (1.13–1.19) < 0.0001	1.15 (1.12–1.18) < 0.0001
7.0–7.8% (53–62 mmol/mol)–Normoalbuminuria and eGFR ≥ 60	1.17 (1.14–1.21) < 0.0001	1.14 (1.10–1.18) < 0.0001	1.13 (1.09–1.17) < 0.0001
7.9–8.7% (63–72 mmol/mol)–Normoalbuminuria and eGFR ≥ 60	1.22 (1.16–1.29) < 0.0001	1.17 (1.11–1.24) < 0.0001	1.19 (1.13–1.26) < 0.0001
8.8–9.6% (73–82 mmol/mol)–Normoalbuminuria and eGFR ≥ 60	1.30 (1.18–1.43) < 0.0001	1.25 (1.14–1.38) < 0.0001	1.25 (1.13–1.38) < 0.0001
≥ 9.7% (≥ 83 mmol/mol)–Normoalbuminuria and eGFR ≥ 60	1.40 (1.22–1.62) < 0.0001	1.35 (1.16–1.56) < 0.0001	1.33 (1.14–1.55) < 0.0002
≤ 6.9% (≤ 52 mmol/mol)–Not Normoalbuminuria or eGFR < 60	1.50 (1.47–1.53) < 0.0001	1.39 (1.36–1.42) < 0.0001	1.38 (1.34–1.41) < 0.0001
7.0–7.8% (53–62 mmol/mol)–Not Normoalbuminuria or eGFR < 60	1.57 (1.52–1.61) < 0.0001	1.45 (1.41–1.49) < 0.0001	1.45 (1.41–1.50) < 0.0001
7.9–8.7% (63–72 mmol/mol)–Not Normoalbuminuria or eGFR < 60	1.75 (1.68–1.81) < 0.0001	1.60 (1.54–1.66) < 0.0001	1.61 (1.55–1.68) < 0.0001
8.8–9.6% (73–82 mmol/mol)–Not Normoalbuminuria or eGFR < 60	1.90 (1.79–2.03) < 0.0001	1.71 (1.60–1.82) < 0.0001	1.72 (1.61–1.84) < 0.0001
≥ 9.7% (≥ 83 mmol/mol)–Not Normoalbuminuria or eGFR < 60	2.03 (1.85–2.23) < 0.0001	1.84 (1.67–2.02) < 0.0001	1.88 (1.71–2.08) < 0.0001

Model 1: adjusted for time-updated age and sex

Model 2: model 1 additionally adjusted for born in Sweden, maximum education level and baseline comorbidities (CHD, HF, VD, stroke, cancer)

Model 3: model 2 additionally adjusted for time-updated diabetes duration

### Risk of atrial fibrillation by 10 mmol/mol higher mean HbA1c in persons with type 2 diabetes

Additionally, the risk of atrial fibrillation was estimated in relation to 1.0% (10.0 mmol/mol) higher mean HbA1c in individuals with type 2 diabetes (Table 5). For individuals with type 2 diabetes, HRs varied between 1.01 and 1.04 in stepwise adjustments for birth in Sweden, education level, baseline comorbidities, and time-updated diabetes duration. When stratified for sex, the risk of atrial fibrillation in men increased by 1.01 (95% CI 1.00–1.03), p = 0.04 , and 1.04 (95% CI 1.03–1.06), p < 0.0001, in women. Further analyses on the impact of time-updated mean HbA1c categories on time to first atrial fibrillation among individuals with type 2 diabetes stratified by sex are shown in Additional file 1: Tables S6 and S7.

**Table 5:** Type 2 diabetes only—all-risk for atrial fibrillation by 1.0% (10 mmol/mol) of time updated mean HbA1c

	P-value	HR	Lower 95% CI	Upper 95% CI
Overall				
Model 1	< 0.0001	1.038	1.029	1.048
Model 2	< 0.0001	1.030	1.021	1.039
Model 3	< 0.0001	1.027	1.017	1.037
Model 3A	< 0.0001	1.010	0.999	1.021
Model 3B	< 0.0001	1.031	1.018	1.043
Model 3C	< 0.0001	1.023	1.005	1.041
Model 3D	< 0.0001	1.013	1.002	1.025
Male				
Model 1	< 0.0001	1.026	1.014	1.038
Model 2	0.0024	1.019	1.007	1.031
Model 3	0.0443	1.013	1.000	1.027
Model 3A	0.4609	0.995	0.981	1.009
Model 3B	0.0070	1.022	1.006	1.037
Model 3C	0.1571	1.017	0.994	1.040
Model 3D	0.4320	0.994	0.980	1.009
Female				
Model 1	< 0.0001	1.054	1.039	1.068
Model 2	< 0.0001	1.044	1.030	1.059
Model 3	< 0.0001	1.044	1.028	1.060
Model 3A	0.0012	1.029	1.011	1.047
Model 3B	< 0.0001	1.043	1.023	1.063
Model 3C	0.0244	1.033	1.004	1.063
Model 3D	< 0.0001	1.040	1.022	1.058

Model 1: adjusted for time-updated age

Model 2: model 1 additionally adjusted for born in Sweden, education level, baseline comorbidities (coronary heart disease, heart failure, valve disease, stroke, cancer)

Model 3: model 2 additionally adjusted for time-updated diabetes duration

Model 3A: model 3 additionally adjusted for time-updated mean systolic blood pressure, time-updated mean body mass index, time updated smoking status, time-updated status about blood pressure lowering medication

Model 3B: model 3 additionally adjusted for time-updated mean high-density lipoprotein, time-updated mean low density lipoprotein, time-updated status about lipid lowering medication

Model 3C: Model 3 additionally adjusted for time-updated insulin method

Model 3D: model 3 additionally adjusted for time-updated albuminuria categories

## Discussion

This large population-based cohort study showed a 35% increased risk of atrial fibrillation in individuals with type 2 diabetes in comparison to matched controls from the general population. The excess risk increased with poorer glycaemic control and severity of renal complications. In individuals with type 2 diabetes, good glycaemic control, and normoalbuminuria, there was a slight yet significantly increased excess risk. The absolute risk of atrial fibrillation was higher for men with type 2 diabetes, but the relative risk was greater for women with type 2 diabetes compared to controls. Women < 55 years with type 2 diabetes had a twofold increased risk, and individuals with type 2 diabetes and CKD stage 5 had a threefold increased risk for atrial fibrillation compared to controls. Additionally, the increased risk of atrial fibrillation in relation to 1.0% (10 mmol/mol) higher mean HbA1c evaluated in persons with diabetes was estimated to range from 1.0 to 4.0%.

Our study is in line with others demonstrating the positive association between diabetes and the risk of atrial fibrillation. In two different meta-analyses including both observational and cohort studies, it was concluded that individuals with type 2 diabetes had a 30.0–40.0% increased risk of atrial fibrillation, even after adjustments for BMI, smoking, hypertension, kidney disease, coronary heart disease, and hyperthyroidism [10, 14]. In our study, women aged < 55 years with type 2 diabetes had an excess risk of atrial fibrillation (HR 2.36) compared to controls, whereas the HR in men in the same age group was 1.78 compared to controls. Older patients with type 2 diabetes had a lower excess but higher absolute risk of atrial fibrillation. A similar pattern was seen in individuals with type 1 diabetes in a previously published cohort study from Sweden [13]. Additionally, a Danish cohort study reported that the risk of atrial fibrillation in individuals with type 2 diabetes was most pronounced in younger patients [15]. When analyzing the excess risk of atrial fibrillation in relation to renal complications, an increased excess risk of atrial fibrillation was observed with greater albuminuria and eGFR. Likewise, in earlier analyses, it has been proposed that hypertension, greater BMI, and albuminuria are risk factors associated with the development of atrial fibrillation in individuals with type 2 diabetes [9, 16, 17]. A study in a Japanese cohort indicated that albuminuria was significantly associated with cardiovascular events and death compared to individuals with type 2 diabetes without renal failure [18]. Additionally, in a large general practice cohort in the United Kingdom including patients with insulin-treated type 2 diabetes, a regression in albuminuria associated with a significant reduction in all-cause mortality was observed [19]. To our knowledge, this is the first study of the excess risk of atrial fibrillation for different HbA1c categories in relation to renal function in comparison with the general population.

Although one should be cautious to make direct comparisons between different studies, it is of interest to notice that the excess risk of heart failure [20], major coronary heart events [21] and stroke [22] has been evaluated in individuals with type 2 diabetes compared to controls from similar cohorts as the current based on the Swedish Diabetes Register. Current together with earlier results therefore give indications how incidences and relative risks differ for various cardiovascular complications. Among individuals with type 2 diabetes, 11.9 cases per 1000 person-years were hospitalized with a diagnosis of heart failure, compared with 6.2 cases per 1000 person-years in controls [20]. The rates of coronary heart disease (CHD) events were 14.6 cases per 1000 person-years among patients with type 2 diabetes versus 8.7 cases per 1000 person-years among controls [21]. Additionally, 10.6 and 6.8 cases per 1000 person-years were diagnosed with stroke among individuals with and without type 2 diabetes, respectively [22]. The corresponding incidence for atrial fibrillation in the current study was 16.0 among individuals with type 2 diabetes compared to 11.9 among controls. Our study shows that atrial fibrillation is a common cardiovascular condition among persons with type 2 diabetes likely somewhat more common than with myocardial infarctions and heart failure.

The risk of atrial fibrillation among individuals with type 2 diabetes was also evaluated in relation to 1.0% (10.0 mmol/mol) higher mean HbA1c. HbA1c had a relatively modest impact on atrial fibrillation with a risk increase of 1.0–4.0% per 10 mmol/mol higher mean HbA1c compared to 12.0–19.0% for heart failure, acute myocardial infarction and CHD death [21, 23]. From these results, it can be concluded that the relative impact of glycaemic control on the risk of atrial fibrillation is relatively low compared with several other cardiovascular complications. From The Action to Control Cardiovascular Risk in Diabetes (ACCORD) trial, it was concluded that participants randomized to an intensive therapeutic strategy did not alter the rate of new-onset atrial fibrillation compared to participants randomized to a standard strategy [24]. Additionally, a study using a Mendelian randomization approach did not observe a causal role between type 2 diabetes and dysglycaemia on the development of atrial fibrillation [25]. However, it has been suggested that HbA1c variability might initiate atrial fibrillation in individuals with type 2 diabetes [26].

Since rates of diabetes mellitus and atrial fibrillation are increasing worldwide and are strong risk factors for stroke and cardiovascular mortality due to thromboembolic risk, it is important to consider early prevention. In a prospective observational study, cardiac rhythm abnormalities were common in patients with moderate to severe CKD not requiring dialysis [27]. Therefore, strategies to prevent renal complications with blood pressure control should be viewed as a goal for protection, both for renal and cardiovascular diseases. For instance, individuals with type 2 diabetes and blood pressure < 130/80 mmHg had a risk of stroke similar to the general population [22]. Also, screening for atrial fibrillation before symptoms are manifested is important in certain patient populations. Clinicians should consider routine screening with pulse palpation or ECG rhythm strip in elderly persons with type 2 diabetes and those with renal complications. This could lead to early initiation of appropriate effective therapy, including antithrombotic therapy, to reduce the risk of stroke and death. Currently, there is no evidence that anti-diabetic therapies with glucagon-like peptide 1 (GLP-1) agonists, sodium-glucose cotransporter-2 (SGLT2) inhibitors nor dipeptidyl peptidase-4 (DPP4) inhibitor affect the development of atrial fibrillation [28].

The link between diabetes mellitus and atrial fibrillation may be explained by several possible mechanisms. The metabolic syndrome characterized by obesity, hypertension, insulin resistance, and dyslipidaemia has been proposed to be associated with increased risk of atrial fibrillation [29]. It has been hypothesized that metabolic syndrome causes a transformation of the epicardial adipose tissue leading to proinflammatory mediators causing endothelial dysfunction, and fibrosis, which in turn leads to the development of structural and electrical atrial remodelling [30–33]. In a nationwide population-based study, body weight status and diabetes had synergistic effects on the risk of new-onset atrial fibrillation [34]. Animal studies confirms that insulin resistance was associated with various aspects of remodelling in the atria, proposing that these might share common electrical and structural remodelling processes in the atria [35].

Strengths of our study include the population-based design including the absolute majority of individuals with type 2 diabetes in Sweden. Additionally, well-known risk factors were available in persons with type 2 diabetes. Limitations include the absence of information on risk factors such as smoking, BMI, blood pressure, and lipid levels in the control population. Another limitation is that we cannot exclude the possibility that atrial fibrillation was detected to a somewhat higher extent in individuals with type 2 diabetes because it is a chronic disease requiring regular visits.

## Conclusion

Type 2 diabetes remains a risk factor for atrial fibrillation, increasing risk by approximately 35% on a group level. Persons with advanced renal complications have substantially higher excess risks with a threefold excess risk at CKD-5. Glycaemic control likely has a relatively limited direct effect on the development of atrial fibrillation, but since it is essential for the development of renal complications it may have an effect over long time periods. Screening for atrial fibrillation should be considered in persons with increased risks including elderly and those with advanced renal complications among persons with type 2 diabetes, especially since the procedure is relatively simple.

## Supplementary information

**Additional file 1: Table S1.** Cox regression for time to atrial fibrillation-overall, men and women. **Table S2.** Adjusted hazard ratios for atrial fibrillation and 95% confidence intervals for time-updated mean HbA1c categories, albuminuria categories and eGFR categories versus the reference group examined by Cox regression–Men. **Table S3.** Adjusted hazard ratios for atrial fibrillation and 95% confidence intervals for time-updated mean HbA1c categories, albuminuria categories and eGFR categories versus the reference group examined by Cox regression–Women. **Table S4.** Adjusted hazard ratios for atrial fribrillation and 95% confidence intervals for time-updated mean HbA1c categories together with albuminuria and eGFR versus the reference group examined by Cox regression–Men. **Table S5.** Adjusted hazard ratios for atrial fibrillation and 95% confidence intervals for time-updated mean HbA1c categories together with albuminuria and eGFR versus the reference group examined by Cox regression–Women. **Table S6.** The impact of time-updated mean HbA1c categories on time to first atrial fibrillation using Cox regression analysis among type 2 diabetes patients–Men. **Table S7.** The impact of time-updated mean HbA1c categories on time to first atrial fibrillation using Cox regression analysis among type 2 diabetes patients–Women.

## Data Availability

Data are not publically available. Data may be available on request to the Swedish National Diabetes Registry.

## Abbreviations

CHD: coronary heart disease
CKD: chronic kidney disease
HbA1c: glycated haemoglobin
HF: heart failure
HR: hazard ratio
IFCC: International Federation of Clinical Chemistry
IRR: incidence risk ratio
LDL: low-density lipoprotein
NDR: The Swedish National Diabetes Registry
NPR: The National Patient Registry
