# Supplementary material for: Risk of atrial fibrillation in persons with type 2 diabetes and the excess risk in relation to glycaemic control and renal function: a Swedish cohort study
Source: Cardiovasc Diabetol. 2020 Jan 18;19:9. doi: 10.1186/s12933-019-0983-1 (PMC6969407; doi:10.1186/s12933-019-0983-1)
Supplement: Supplementary file 1 — Additional file 1: Table S1. Cox regression for time to atrial fibrillation-overall, men and women. Table S2. Adjusted hazard ratios for atrial fibrillation and 95% confidence intervals for time-updated mean HbA1c categories, albuminuria categories and eGFR categories versus the reference group examined by Cox regression–Men. Table S3. Adjusted hazard ratios for atrial fibrillation and 95% confidence intervals for time-updated mean HbA1c categories, albuminuria categories and eGFR categories versus the reference group examined by Cox regression–Women. Table S4. Adjusted hazard ratios for atrial fribrillation and 95% confidence intervals for time-updated mean HbA1c categories together with albuminuria and eGFR versus the reference group examined by Cox regression–Men. Table S5. Adjusted hazard ratios for atrial fibrillation and 95% confidence intervals for time-updated mean HbA1c categories together with albuminuria and eGFR versus the reference group examined by Cox regression–Women. Table S6. The impact of time-updated mean HbA1c categories on time to first atrial fibrillation using Cox regression analysis among type 2 diabetes patients–Men. Table S7. The impact of time-updated mean HbA1c categories on time to first atrial fibrillation using Cox regression analysis among type 2 diabetes patients–Women. [file 12933_2019_983_MOESM1_ESM.docx]

**Supplementary appendix**

Supplement to:

Risk of atrial fibrillation in persons with type 2 diabetes and the excess risk in relation to glycaemic control and renal function: a Swedish cohort study

Shilan S Ahmadi MD^1,2^, Ann-Marie Svensson PhD^1,3^, Aldina Pivodic M.Sc^4,5^, Annika Rosengren MD, PhD^1,6^, Marcus Lind MD, PhD^1,2^

^1^ Department of Molecular and Clinical Medicine, University of Gothenburg, Gothenburg, Sweden

^2^ Department of Medicine, NU-Hospital Group, Trollhättan/Uddevalla, Sweden

^3^ Swedish National Diabetes Register, Centre of Registers, Gothenburg, Sweden.

^4^ Statistiska Konsultgruppen, Gothenburg, Sweden

^5^ Department of Ophthalmology, Institute of Neuroscience and Physiology, Sahlgrenska Academy, University of Gothenburg, Gothenburg, Sweden

^6^ Sahlgrenska University Hospital, Gothenburg, Sweden

1. Table of contents

[2.1 ICD Codes 3](#_Toc18169907)

[2.2 Table S1. Cox regression for time to atrial fibrillation - overall, men and women. 4](#_Toc18169908)

[2.3 Table S2. Adjusted hazard ratios for atrial fibrillation and 95% confidence intervals for time-updated mean HbA1c categories, albuminuria categories and eGFR categories versus the reference group examined by Cox regression – Men 6](#_Toc18169909)

[2.4 Table S3. Adjusted hazard ratios for atrial fibrillation and 95% confidence intervals for time-updated mean HbA1c categories, albuminuria categories and eGFR categories versus the reference group examined by Cox regression – Women 7](#_Toc18169910)

[2.5 Table S4. Adjusted hazard ratios for atrial fribrillation and 95% confidence intervals for time-updated mean HbA1c categories together with albuminuria and eGFR versus the reference group examined by Cox regression - Men 8](#_Toc18169911)

[2.6 Table S5. Adjusted hazard ratios for atrial fibrillation and 95% confidence intervals for time-updated mean HbA1c categories together with albuminuria and eGFR versus the reference group examined by Cox regression – Women 10](#_Toc18169912)

[2.7 Table S6. The impact of time-updated mean HbA1c categories on time to first atrial fibrillation using Cox regression analysis among type 2 diabetes patients – Men 12](#_Toc18169913)

[2.8 Table S7. The impact of time-updated mean HbA1c categories on time to first atrial fibrillation using Cox regression analysis among type 2 diabetes patients – Women 13](#_Toc18169914)

**2. Tables**

### **2.1 ICD Codes**

The National patient register (NPR) includes the Inpatient register and the Outpatient register and includes information on principal and contributory diagnoses from inpatient and outpatient hospital discharge diagnoses, deaths and causes of death. The NPR have a nationwide coverage.

The following ICD-9 and ICD-10 codes were collected:

Coronary heart disease 410-414 (ICD-9), I20-I25 (ICD-10)

Acute myocardial infarction 410 (ICD-9) and I21 (ICD-10)

Main and sub-diagnoses from the inpatient register;

Stroke 431-434,436 (ICD-9) and I61-I64 (ICD-10)

Hospitalization for heart failure 428 (ICD-9) and I50 (ICD-10)

Atrial fibrillation 427 D(ICD-9) and I.48 (ICD-10)

Main and sub-diagnoses from both inpatient and outpatient register;

Cancer 140-208 (ICD-9) and C00-C097 (ICD-10)

### **2.2 Table S1. Cox regression for time to atrial fibrillation - overall, men and women.**

**Overall – Cox Regression for Time to atrial fibrillation**

|  | **p-value** | **HR** | **Lower 95% CI** | **Upper 95% CI** |
| --- | --- | --- | --- | --- |
| **Model 1** | <.0001 | 1.366 | 1.350 | 1.381 |
| **Model 2** | <.0001 | 1.295 | 1.280 | 1.310 |
| **Model 3 DD=1yr** | <.0001 | 1.260 | 1.239 | 1.282 |
| **Model 3 DD=3yr** | <.0001 | 1.266 | 1.247 | 1.286 |
| **Model 3 DD=7yr** | <.0001 | 1.278 | 1.262 | 1.295 |
| **Model 3 DD=13yr** | <.0001 | 1.297 | 1.281 | 1.313 |
| **Model 3 DD=19yr** | <.0001 | 1.315 | 1.295 | 1.336 |
| Model 1: adjusted for time-updated age and sex  Model 2: model 1 additionally adjusted for country of birth, education and baseline comorbidities  Model 3: model 2 additionally adjusted for time-updated diabetes duration and effects shown for pctl 10, 25, 50, 75, 90. | | | | |

**Male – Cox Regression for Time to atrial fibrillation**

|  | **p-value** | **HR** | **Lower 95% CI** | **Upper 95% CI** |
| --- | --- | --- | --- | --- |
| **Model 1** | <.0001 | 1.343 | 1.324 | 1.364 |
| **Model 2** | <.0001 | 1.278 | 1.259 | 1.297 |
| **Model 3 DD=1yr** | <.0001 | 1.243 | 1.215 | 1.271 |
| **Model 3 DD=3yr** | <.0001 | 1.248 | 1.223 | 1.273 |
| **Model 3 DD=7yr** | <.0001 | 1.258 | 1.237 | 1.279 |
| **Model 3 DD=13yr** | <.0001 | 1.273 | 1.253 | 1.294 |
| **Model 3 DD=19yr** | <.0001 | 1.288 | 1.262 | 1.316 |
| Model 1: adjusted for time-updated age  Model 2: model 1 additionally adjusted for country of birth, education and baseline comorbidities  Model 3: model 2 additionally adjusted for time-updated diabetes duration and effects shown for pctl 10, 25, 50, 75, 90. | | | | |

**Female – Cox Regression for Time to atrial fibrillation**

|  | **p-value** | **HR** | **Lower 95% CI** | **Upper 95% CI** |
| --- | --- | --- | --- | --- |
| **Model 1** | <.0001 | 1.399 | 1.375 | 1.423 |
| **Model 2** | <.0001 | 1.321 | 1.297 | 1.345 |
| **Model 3 DD=1yr** | <.0001 | 1.289 | 1.254 | 1.324 |
| **Model 3 DD=3yr** | <.0001 | 1.296 | 1.264 | 1.328 |
| **Model 3 DD=7yr** | <.0001 | 1.310 | 1.284 | 1.337 |
| **Model 3 DD=13yr** | <.0001 | 1.332 | 1.307 | 1.358 |
| **Model 3 DD=19yr** | <.0001 | 1.355 | 1.322 | 1.388 |
| Model 1: adjusted for time-updated age  Model 2: model 1 additionally adjusted for country of birth, education and baseline comorbidities  Model 3: model 2 additionally adjusted for time-updated diabetes duration and effects shown for pctl 10, 25, 50, 75, 90. | | | | |

### **2.3 Table S2. Adjusted hazard ratios for atrial fibrillation and 95% confidence intervals for time-updated mean HbA1c categories, albuminuria categories and eGFR categories versus the reference group examined by Cox regression – Men**

|  | **Hazard ratio (95% CI) p-value** | | |
| --- | --- | --- | --- |
| **Atrial fibrillation** | **Model 1** | **Model 2** | **Model 3** |
| **Time updated mean HbA1c categories** | n events=107398 N subjects=1384149 data used = 99.7% | n events=105779 N subjects=1362613 data used = 98.1% | n events=104284 N subjects=1345102 data used = 96.9% |
| **Controls (reference)** | 1.00 | 1.00 | 1.00 |
| **<=6.9% (<=52 mmol/mol)** | 1.31 (1.28 - 1.33) <.0001 | 1.25 (1.22 - 1.27) <.0001 | 1.23 (1.21 - 1.26) <.0001 |
| **7.0-7.8% (53-62 mmol/mol)** | 1.33 (1.30 - 1.37) <.0001 | 1.27 (1.24 - 1.30) <.0001 | 1.25 (1.21 - 1.29) <.0001 |
| **7.9-8.7% (63-72 mmol/mol)** | 1.42 (1.36 - 1.47) <.0001 | 1.34 (1.29 - 1.39) <.0001 | 1.33 (1.28 - 1.39) <.0001 |
| **8.8-9.6% (73-82 mmol/mol)** | 1.55 (1.46 - 1.65) <.0001 | 1.43 (1.34 - 1.53) <.0001 | 1.42 (1.33 - 1.52) <.0001 |
| **>=9.7% (>=83 mmol/mol)** | 1.59 (1.45 - 1.74) <.0001 | 1.50 (1.37 - 1.64) <.0001 | 1.48 (1.34 - 1.62) <.0001 |
| **Time updated albuminuria categories** | n events=103924 N subjects=1346827 data used = 97.0% | n events=102378 N subjects=1326252 data used = 95.5% | n events=101297 N subjects=1314558 data used = 94.7% |
| **Controls (reference)** | 1.00 | 1.00 | 1.00 |
| **Normoalbuminuria** | 1.19 (1.17 - 1.21) <.0001 | 1.14 (1.12 - 1.17) <.0001 | 1.13 (1.11 - 1.16) <.0001 |
| **Microalbuminuria** | 1.57 (1.52 - 1.62) <.0001 | 1.49 (1.44 - 1.54) <.0001 | 1.48 (1.43 - 1.53) <.0001 |
| **Macroalbuminuria** | 1.88 (1.81 - 1.95) <.0001 | 1.74 (1.67 - 1.80) <.0001 | 1.73 (1.66 - 1.80) <.0001 |
| **CKD stage 5** | 3.62 (3.13 - 4.20) <.0001 | 3.13 (2.70 - 3.64) <.0001 | 3.15 (2.70 - 3.68) <.0001 |
| **Time updated eGFR categories** | n events=105847 N subjects=1373954 data used = 98.9% | n events=104261 N subjects=1352756 data used = 97.4% | n events=102828 N subjects=1335932 data used = 96.2% |
| **Controls (reference)** | 1.00 | 1.00 | 1.00 |
| **CKD stage 1 (eGFR >=90)** | 1.35 (1.31 - 1.40) <.0001 | 1.33 (1.28 - 1.37) <.0001 | 1.31 (1.27 - 1.36) <.0001 |
| **CKD stage 2 (eGFR 60-89)** | 1.26 (1.24 - 1.29) <.0001 | 1.22 (1.19 - 1.24) <.0001 | 1.20 (1.18 - 1.23) <.0001 |
| **CKD stage 3 (eGFR 30-59)** | 1.45 (1.41 - 1.49) <.0001 | 1.32 (1.28 - 1.36) <.0001 | 1.30 (1.26 - 1.34) <.0001 |
| **CKD stage 4 (eGFR 15-29)** | 2.16 (1.99 - 2.34) <.0001 | 1.83 (1.69 - 1.98) <.0001 | 1.82 (1.67 - 1.98) <.0001 |
| **CKD stage 5 (eGFR <15 or dialysis)** | 3.67 (3.16 - 4.26) <.0001 | 3.17 (2.73 - 3.68) <.0001 | 3.15 (2.70 - 3.67) <.0001 |
| Model 1: adjusted for time-updated age Model 2: Model 1 additionally adjusted for born in Sweden, maximum education level and baseline comorbidities (CHD, HF, VD, stroke, cancer) Model 3: Model 2 additionally adjusted for time-updated diabetes duration (effects taken for DD=7 years [median]). | | | |

### **2.4 Table S3. Adjusted hazard ratios for atrial fibrillation and 95% confidence intervals for time-updated mean HbA1c categories, albuminuria categories and eGFR categories versus the reference group examined by Cox regression – Women**

|  | **Hazard ratio (95% CI) p-value** | | |
| --- | --- | --- | --- |
| **Atrial fibrillation** | **Model 1** | **Model 2** | **Model 3** |
| **Time updated mean HbA1c categories** | n events=78395 N subjects=1159954 data used = 99.6% | n events=76302 N subjects=1136114 data used = 97.6% | n events=75161 N subjects=1120438 data used = 96.2% |
| **Controls (reference)** | 1.00 | 1.00 | 1.00 |
| **<=6.9% (<=52 mmol/mol)** | 1.33 (1.30 - 1.36) <.0001 | 1.27 (1.24 - 1.30) <.0001 | 1.26 (1.23 - 1.29) <.0001 |
| **7.0-7.8% (53-62 mmol/mol)** | 1.41 (1.37 - 1.46) <.0001 | 1.33 (1.29 - 1.37) <.0001 | 1.33 (1.28 - 1.37) <.0001 |
| **7.9-8.7% (63-72 mmol/mol)** | 1.56 (1.49 - 1.63) <.0001 | 1.44 (1.38 - 1.51) <.0001 | 1.45 (1.38 - 1.52) <.0001 |
| **8.8-9.6% (73-82 mmol/mol)** | 1.70 (1.58 - 1.83) <.0001 | 1.58 (1.46 - 1.70) <.0001 | 1.54 (1.42 - 1.67) <.0001 |
| **>=9.7% (>=83 mmol/mol)** | 1.74 (1.57 - 1.94) <.0001 | 1.66 (1.49 - 1.84) <.0001 | 1.70 (1.52 - 1.90) <.0001 |
| **Time updated albuminuria categories** | n events=75456 N subjects=1126983 data used = 96.8% | n events=73512 N subjects=1104757 data used = 94.9% | n events=72762 N subjects=1094921 data used = 94.0% |
| **Controls (reference)** | 1.00 | 1.00 | 1.00 |
| **Normoalbuminuria** | 1.30 (1.27 - 1.33) <.0001 | 1.24 (1.21 - 1.27) <.0001 | 1.23 (1.20 - 1.26) <.0001 |
| **Microalbuminuria** | 1.75 (1.68 - 1.82) <.0001 | 1.63 (1.56 - 1.70) <.0001 | 1.61 (1.54 - 1.69) <.0001 |
| **Macroalbuminuria** | 2.00 (1.89 - 2.11) <.0001 | 1.78 (1.68 - 1.89) <.0001 | 1.77 (1.67 - 1.89) <.0001 |
| **CKD stage 5** | 3.96 (3.26 - 4.79) <.0001 | 3.41 (2.81 - 4.15) <.0001 | 3.41 (2.78 - 4.19) <.0001 |
| **Time updated eGFR categories** | n events=77260 N subjects=1151603 data used = 98.9% | n events=75221 N subjects=1128262 data used = 96.9% | n events=74130 N subjects=1113157 data used = 95.6% |
| **Controls (reference)** | 1.00 | 1.00 | 1.00 |
| **CKD stage 1 (eGFR >=90)** | 1.40 (1.33 - 1.48) <.0001 | 1.37 (1.29 - 1.44) <.0001 | 1.35 (1.28 - 1.43) <.0001 |
| **CKD stage 2 (eGFR 60-89)** | 1.31 (1.27 - 1.34) <.0001 | 1.25 (1.22 - 1.29) <.0001 | 1.25 (1.21 - 1.28) <.0001 |
| **CKD stage 3 (eGFR 30-59)** | 1.49 (1.45 - 1.53) <.0001 | 1.37 (1.33 - 1.41) <.0001 | 1.36 (1.32 - 1.40) <.0001 |
| **CKD stage 4 (eGFR 15-29)** | 2.03 (1.87 - 2.19) <.0001 | 1.70 (1.57 - 1.85) <.0001 | 1.68 (1.55 - 1.83) <.0001 |
| **CKD stage 5 (eGFR <15 or dialysis)** | 4.00 (3.29 - 4.85) <.0001 | 3.46 (2.84 - 4.21) <.0001 | 3.42 (2.79 - 4.20) <.0001 |
| Model 1: adjusted for time-updated age Model 2: Model 1 additionally adjusted for born in Sweden, maximum education level and baseline comorbidities (CHD, HF, VD, stroke, cancer) Model 3: Model 2 additionally adjusted for time-updated diabetes duration (effects taken for DD=7 years [median]). | | | |

### **2.5 Table S4. Adjusted hazard ratios for atrial fibrillation and 95% confidence intervals for time-updated mean HbA1c categories together with albuminuria and eGFR versus the reference group examined by Cox regression - Men**

|  | **Hazard ratio (95% CI) p-value** | | |
| --- | --- | --- | --- |
| **Atrial fibrillation** | **Model 1** | **Model 2** | **Model 3** |
| **Time updated mean HbA1c categories and albuminuria** | n events=103795 N subjects=1345856 data used = 96.9% | n events=102251 N subjects=1325311 data used = 95.4% | n events=101200 N subjects=1313869 data used = 94.6% |
| **Controls (reference)** | 1.00 | 1.00 | 1.00 |
| **<=6.9% (<=52 mmol/mol) - Normoalbuminuria** | 1.19 (1.16 - 1.22) <.0001 | 1.15 (1.12 - 1.18) <.0001 | 1.14 (1.11 - 1.17) <.0001 |
| **7.0-7.8% (53-62 mmol/mol) - Normoalbuminuria** | 1.16 (1.12 - 1.21) <.0001 | 1.11 (1.07 - 1.16) <.0001 | 1.11 (1.06 - 1.15) <.0001 |
| **7.9-8.7% (63-72 mmol/mol) - Normoalbuminuria** | 1.21 (1.14 - 1.29) <.0001 | 1.16 (1.09 - 1.23) <.0001 | 1.17 (1.10 - 1.24) <.0001 |
| **8.8-9.6% (73-82 mmol/mol) - Normoalbuminuria** | 1.26 (1.14 - 1.40) <.0001 | 1.19 (1.07 - 1.32) 0.0014 | 1.18 (1.05 - 1.31) 0.0036 |
| **>=9.7% (>=83 mmol/mol) - Normoalbuminuria** | 1.30 (1.11 - 1.52) 0.0013 | 1.21 (1.03 - 1.42) 0.020 | 1.18 (1.00 - 1.40) 0.051 |
| **<=6.9% (<=52 mmol/mol) - Not Normoalbuminuria** | 1.68 (1.62 - 1.74) <.0001 | 1.57 (1.51 - 1.63) <.0001 | 1.55 (1.49 - 1.61) <.0001 |
| **7.0-7.8% (53-62 mmol/mol) - Not Normoalbuminuria** | 1.66 (1.59 - 1.73) <.0001 | 1.57 (1.50 - 1.63) <.0001 | 1.56 (1.49 - 1.63) <.0001 |
| **7.9-8.7% (63-72 mmol/mol) - Not Normoalbuminuria** | 1.76 (1.66 - 1.86) <.0001 | 1.65 (1.55 - 1.75) <.0001 | 1.65 (1.55 - 1.76) <.0001 |
| **8.8-9.6% (73-82 mmol/mol) - Not Normoalbuminuria** | 2.00 (1.83 - 2.19) <.0001 | 1.83 (1.66 - 2.00) <.0001 | 1.83 (1.66 - 2.01) <.0001 |
| **>=9.7% (>=83 mmol/mol) - Not Normoalbuminuria** | 2.01 (1.75 - 2.31) <.0001 | 1.85 (1.61 - 2.13) <.0001 | 1.87 (1.62 - 2.16) <.0001 |
| **Time updated mean HbA1c categories and eGFR** | n events=105649 N subjects=1372287 data used = 98.8% | n events=104065 N subjects=1351128 data used = 97.3% | n events=102699 N subjects=1334886 data used = 96.1% |
| **Controls (reference)** | 1.00 | 1.00 | 1.00 |
| **<=6.9% (<=52 mmol/mol) - eGFR>=60** | 1.26 (1.23 - 1.29) <.0001 | 1.22 (1.19 - 1.25) <.0001 | 1.21 (1.18 - 1.24) <.0001 |
| **7.0-7.8% (53-62 mmol/mol) - eGFR>=60** | 1.26 (1.22 - 1.30) <.0001 | 1.22 (1.18 - 1.26) <.0001 | 1.21 (1.17 - 1.25) <.0001 |
| **7.9-8.7% (63-72 mmol/mol) - eGFR>=60** | 1.36 (1.29 - 1.43) <.0001 | 1.31 (1.25 - 1.38) <.0001 | 1.31 (1.25 - 1.38) <.0001 |
| **8.8-9.6% (73-82 mmol/mol) - eGFR>=60** | 1.47 (1.36 - 1.60) <.0001 | 1.40 (1.29 - 1.52) <.0001 | 1.39 (1.28 - 1.52) <.0001 |
| **>=9.7% (>=83 mmol/mol) - eGFR>=60** | 1.54 (1.37 - 1.72) <.0001 | 1.48 (1.32 - 1.66) <.0001 | 1.48 (1.31 - 1.67) <.0001 |
| **<=6.9% (<=52 mmol/mol) - eGFR<60** | 1.47 (1.41 - 1.52) <.0001 | 1.33 (1.28 - 1.38) <.0001 | 1.31 (1.25 - 1.36) <.0001 |
| **7.0-7.8% (53-62 mmol/mol) - eGFR<60** | 1.56 (1.49 - 1.63) <.0001 | 1.42 (1.35 - 1.48) <.0001 | 1.40 (1.33 - 1.47) <.0001 |
| **7.9-8.7% (63-72 mmol/mol) - eGFR<60** | 1.61 (1.50 - 1.73) <.0001 | 1.44 (1.34 - 1.55) <.0001 | 1.45 (1.34 - 1.56) <.0001 |
| **8.8-9.6% (73-82 mmol/mol) - eGFR<60** | 1.80 (1.60 - 2.03) <.0001 | 1.55 (1.37 - 1.76) <.0001 | 1.59 (1.40 - 1.80) <.0001 |
| **>=9.7% (>=83 mmol/mol) - eGFR<60** | 1.78 (1.47 - 2.16) <.0001 | 1.54 (1.27 - 1.87) <.0001 | 1.55 (1.27 - 1.90) <.0001 |
| **Time updated mean HbA1c categories albuminuria and eGFR** | n events=103981 N subjects=1348598 data used = 97.1% | n events=102427 N subjects=1327968 data used = 95.6% | n events=101300 N subjects=1315808 data used = 94.7% |
| **Controls (reference)** | 1.00 | 1.00 | 1.00 |
| **<=6.9% (<=52 mmol/mol) - Normoalbuminuria and eGFR>=60** | 1.17 (1.14 - 1.21) <.0001 | 1.14 (1.11 - 1.18) <.0001 | 1.13 (1.10 - 1.17) <.0001 |
| **7.0-7.8% (53-62 mmol/mol) - Normoalbuminuria and eGFR>=60** | 1.13 (1.08 - 1.18) <.0001 | 1.09 (1.04 - 1.14) <.0001 | 1.08 (1.04 - 1.13) 0.0005 |
| **7.9-8.7% (63-72 mmol/mol) - Normoalbuminuria and eGFR>=60** | 1.17 (1.09 - 1.25) <.0001 | 1.13 (1.05 - 1.21) 0.0006 | 1.15 (1.07 - 1.23) 0.0002 |
| **8.8-9.6% (73-82 mmol/mol) - Normoalbuminuria and eGFR>=60** | 1.23 (1.09 - 1.39) 0.0011 | 1.18 (1.04 - 1.33) 0.012 | 1.17 (1.03 - 1.33) 0.017 |
| **>=9.7% (>=83 mmol/mol) - Normoalbuminuria and eGFR>=60** | 1.26 (1.04 - 1.52) 0.018 | 1.19 (0.98 - 1.44) 0.086 | 1.15 (0.94 - 1.41) 0.18 |
| **<=6.9% (<=52 mmol/mol) - Not Normoalbuminuria or eGFR<60** | 1.52 (1.48 - 1.57) <.0001 | 1.41 (1.36 - 1.45) <.0001 | 1.39 (1.34 - 1.43) <.0001 |
| **7.0-7.8% (53-62 mmol/mol) - Not Normoalbuminuria or eGFR<60** | 1.56 (1.50 - 1.62) <.0001 | 1.45 (1.40 - 1.50) <.0001 | 1.44 (1.39 - 1.50) <.0001 |
| **7.9-8.7% (63-72 mmol/mol) - Not Normoalbuminuria or eGFR<60** | 1.66 (1.58 - 1.75) <.0001 | 1.54 (1.46 - 1.62) <.0001 | 1.55 (1.46 - 1.64) <.0001 |
| **8.8-9.6% (73-82 mmol/mol) - Not Normoalbuminuria or eGFR<60** | 1.86 (1.71 - 2.02) <.0001 | 1.67 (1.53 - 1.82) <.0001 | 1.69 (1.55 - 1.85) <.0001 |
| **>=9.7% (>=83 mmol/mol) - Not Normoalbuminuria or eGFR<60** | 1.89 (1.67 - 2.15) <.0001 | 1.73 (1.52 - 1.96) <.0001 | 1.76 (1.54 - 2.00) <.0001 |
| Model 1: adjusted for time-updated age Model 2: Model 1 additionally adjusted for born in Sweden, maximum education level and baseline comorbidities (CHD, HF, VD, stroke, cancer) Model 3: Model 2 additionally adjusted for time-updated diabetes duration. | | | |

### **2.6 Table S5. Adjusted hazard ratios for atrial fibrillation and 95% confidence intervals for time-updated mean HbA1c categories together with albuminuria and eGFR versus the reference group examined by Cox regression – Women**

|  | **Hazard ratio (95% CI) p-value** | | |
| --- | --- | --- | --- |
| **Atrial fibrillation** | **Model 1** | **Model 2** | **Model 3** |
| **Time updated mean HbA1c categories and albuminuria** | n events=75371 N subjects=1126126 data used = 96.7% | n events=73432 N subjects=1103950 data used = 94.8% | n events=72699 N subjects=1094374 data used = 94.0% |
| **Controls (reference)** | 1.00 | 1.00 | 1.00 |
| **<=6.9% (<=52 mmol/mol) - Normoalbuminuria** | 1.26 (1.22 - 1.30) <.0001 | 1.21 (1.17 - 1.25) <.0001 | 1.20 (1.17 - 1.24) <.0001 |
| **7.0-7.8% (53-62 mmol/mol) - Normoalbuminuria** | 1.29 (1.24 - 1.35) <.0001 | 1.23 (1.18 - 1.28) <.0001 | 1.24 (1.18 - 1.29) <.0001 |
| **7.9-8.7% (63-72 mmol/mol) - Normoalbuminuria** | 1.40 (1.32 - 1.49) <.0001 | 1.31 (1.23 - 1.39) <.0001 | 1.33 (1.24 - 1.42) <.0001 |
| **8.8-9.6% (73-82 mmol/mol) - Normoalbuminuria** | 1.58 (1.41 - 1.75) <.0001 | 1.50 (1.35 - 1.67) <.0001 | 1.49 (1.33 - 1.67) <.0001 |
| **>=9.7% (>=83 mmol/mol) - Normoalbuminuria** | 1.62 (1.37 - 1.91) <.0001 | 1.53 (1.29 - 1.81) <.0001 | 1.58 (1.33 - 1.87) <.0001 |
| **<=6.9% (<=52 mmol/mol) - Not Normoalbuminuria** | 1.70 (1.61 - 1.79) <.0001 | 1.56 (1.48 - 1.65) <.0001 | 1.56 (1.47 - 1.65) <.0001 |
| **7.0-7.8% (53-62 mmol/mol) - Not Normoalbuminuria** | 1.87 (1.76 - 1.99) <.0001 | 1.74 (1.63 - 1.85) <.0001 | 1.74 (1.63 - 1.86) <.0001 |
| **7.9-8.7% (63-72 mmol/mol) - Not Normoalbuminuria** | 2.09 (1.93 - 2.26) <.0001 | 1.89 (1.74 - 2.05) <.0001 | 1.89 (1.74 - 2.07) <.0001 |
| **8.8-9.6% (73-82 mmol/mol) - Not Normoalbuminuria** | 2.12 (1.87 - 2.40) <.0001 | 1.88 (1.65 - 2.15) <.0001 | 1.90 (1.66 - 2.18) <.0001 |
| **>=9.7% (>=83 mmol/mol) - Not Normoalbuminuria** | 2.56 (2.16 - 3.05) <.0001 | 2.27 (1.90 - 2.72) <.0001 | 2.35 (1.96 - 2.82) <.0001 |
| **Time updated mean HbA1c categories and eGFR** | n events=77094 N subjects=1149923 data used = 98.8% | n events=75062 N subjects=1126657 data used = 96.8% | n events=74034 N subjects=1112199 data used = 95.5% |
| **Controls (reference)** | 1.00 | 1.00 | 1.00 |
| **<=6.9% (<=52 mmol/mol) - eGFR>=60** | 1.27 (1.24 - 1.31) <.0001 | 1.23 (1.19 - 1.27) <.0001 | 1.22 (1.18 - 1.27) <.0001 |
| **7.0-7.8% (53-62 mmol/mol) - eGFR>=60** | 1.34 (1.29 - 1.40) <.0001 | 1.29 (1.23 - 1.35) <.0001 | 1.29 (1.23 - 1.35) <.0001 |
| **7.9-8.7% (63-72 mmol/mol) - eGFR>=60** | 1.42 (1.33 - 1.52) <.0001 | 1.35 (1.26 - 1.44) <.0001 | 1.36 (1.26 - 1.46) <.0001 |
| **8.8-9.6% (73-82 mmol/mol) - eGFR>=60** | 1.56 (1.40 - 1.74) <.0001 | 1.47 (1.31 - 1.64) <.0001 | 1.44 (1.28 - 1.62) <.0001 |
| **>=9.7% (>=83 mmol/mol) - eGFR>=60** | 1.57 (1.35 - 1.84) <.0001 | 1.56 (1.33 - 1.84) <.0001 | 1.60 (1.35 - 1.89) <.0001 |
| **<=6.9% (<=52 mmol/mol) - eGFR<60** | 1.44 (1.39 - 1.50) <.0001 | 1.33 (1.28 - 1.38) <.0001 | 1.33 (1.28 - 1.38) <.0001 |
| **7.0-7.8% (53-62 mmol/mol) - eGFR<60** | 1.53 (1.46 - 1.61) <.0001 | 1.40 (1.33 - 1.47) <.0001 | 1.40 (1.33 - 1.47) <.0001 |
| **7.9-8.7% (63-72 mmol/mol) - eGFR<60** | 1.84 (1.72 - 1.97) <.0001 | 1.65 (1.54 - 1.76) <.0001 | 1.67 (1.56 - 1.80) <.0001 |
| **8.8-9.6% (73-82 mmol/mol) - eGFR<60** | 1.94 (1.73 - 2.17) <.0001 | 1.73 (1.54 - 1.94) <.0001 | 1.71 (1.51 - 1.93) <.0001 |
| **>=9.7% (>=83 mmol/mol) - eGFR<60** | 2.17 (1.83 - 2.57) <.0001 | 1.86 (1.56 - 2.22) <.0001 | 1.93 (1.61 - 2.32) <.0001 |
| **Time updated mean HbA1c categories albuminuria and eGFR** | n events=75999 N subjects=1131619 data used = 97.2% | n events=74032 N subjects=1109190 data used = 95.3% | n events=73166 N subjects=1098284 data used = 94.3% |
| **Controls (reference)** | 1.00 | 1.00 | 1.00 |
| **<=6.9% (<=52 mmol/mol) - Normoalbuminuria and eGFR>=60** | 1.22 (1.18 - 1.27) <.0001 | 1.19 (1.15 - 1.24) <.0001 | 1.18 (1.13 - 1.23) <.0001 |
| **7.0-7.8% (53-62 mmol/mol) - Normoalbuminuria and eGFR>=60** | 1.26 (1.19 - 1.32) <.0001 | 1.22 (1.15 - 1.28) <.0001 | 1.22 (1.15 - 1.29) <.0001 |
| **7.9-8.7% (63-72 mmol/mol) - Normoalbuminuria and eGFR>=60** | 1.30 (1.20 - 1.42) <.0001 | 1.24 (1.14 - 1.35) <.0001 | 1.27 (1.16 - 1.39) <.0001 |
| **8.8-9.6% (73-82 mmol/mol) - Normoalbuminuria and eGFR>=60** | 1.42 (1.22 - 1.65) <.0001 | 1.38 (1.18 - 1.61) <.0001 | 1.37 (1.17 - 1.61) <.0001 |
| **>=9.7% (>=83 mmol/mol) - Normoalbuminuria and eGFR>=60** | 1.64 (1.32 - 2.04) <.0001 | 1.61 (1.29 - 2.00) <.0001 | 1.64 (1.31 - 2.05) <.0001 |
| **<=6.9% (<=52 mmol/mol) - Not Normoalbuminuria or eGFR<60** | 1.47 (1.43 - 1.52) <.0001 | 1.36 (1.32 - 1.41) <.0001 | 1.36 (1.32 - 1.41) <.0001 |
| **7.0-7.8% (53-62 mmol/mol) - Not Normoalbuminuria or eGFR<60** | 1.58 (1.51 - 1.65) <.0001 | 1.45 (1.39 - 1.51) <.0001 | 1.46 (1.39 - 1.53) <.0001 |
| **7.9-8.7% (63-72 mmol/mol) - Not Normoalbuminuria or eGFR<60** | 1.86 (1.75 - 1.97) <.0001 | 1.67 (1.58 - 1.77) <.0001 | 1.70 (1.60 - 1.82) <.0001 |
| **8.8-9.6% (73-82 mmol/mol) - Not Normoalbuminuria or eGFR<60** | 1.96 (1.78 - 2.16) <.0001 | 1.75 (1.58 - 1.93) <.0001 | 1.76 (1.58 - 1.95) <.0001 |
| **>=9.7% (>=83 mmol/mol) - Not Normoalbuminuria or eGFR<60** | 2.21 (1.92 - 2.54) <.0001 | 1.98 (1.71 - 2.28) <.0001 | 2.05 (1.77 - 2.38) <.0001 |
| Model 1: adjusted for time-updated age Model 2: Model 1 additionally adjusted for born in Sweden, maximum education level and baseline comorbidities (CHD, HF, VD, stroke, cancer) Model 3: Model 2 additionally adjusted for time-updated diabetes duration. | | | |

### **2.7 Table S6. The impact of time-updated mean HbA1c categories on time to first atrial fibrillation using Cox regression analysis among type 2 diabetes patients – Men**

|  | **Hazard ratio (95% CI) p-value** | | | | | | |
| --- | --- | --- | --- | --- | --- | --- | --- |
| **Time to first atrial fibrillation** | **Model 1** | **Model 2** | **Model 3** | **Model 3A** | **Model 3B** | **Model 3C** | **Model 3D** |
| **Time updated mean HbA1c categories** |  |  |  |  |  |  |  |
| **<=6.9% (<=52 mmol/mol)** | 1.00 | 1.00 | 1.00 |  |  |  |  |
| **7.0-7.8% (53-62 mmol/mol)** | 1.01 (0.98- 1.05)  0.38 | 1.01 (0.98-1.04)  0.66 | 1.00 (0.96-1.03)  0.81 | 0.97 (0.94-1.01)  0.11 | 1.01 (0.97-1.04)  0.79 | 0.96 (0.90-1.02)  0.22 | 0.96 (0.93-1.00)  0.052 |
| **7.9-8.7% (63-72 mmol/mol)** | 1.07 (1.02-1.11)  0.0037 | 1.05 (1.01-1.10)  0.027 | 1.04 (0.99-1.09)  0.094 | 1.00 (0.95-1.04)  0.83 | 1.05 (1.00-1.11)  0.051 | 1.02 (0.95-1.10)  0.52 | 0.99 (0.94-1.04)  0.74 |
| **8.8-9.6% (73-82 mmol/mol)** | 1.15 (1.08-1.23)  <.0001 | 1.11 (1.03-1.18)  0.0032 | 1.09 (1.02-1.17)  0.012 | 1.02 (0.95-1.10)  0.53 | 1.14 (1.05-1.23)  0.0022 | 1.00 (0.91-1.11)  0.99 | 1.02 (0.95-1.10)  0.55 |
| **>=9.7% (>=83 mmol/mol)** | 1.16 (1.06-1.27)  0.0017 | 1.13 (1.03-1.24)  0.0075 | 1.11 (1.01-1.22)  0.033 | 1.06 (0.95-1.18)  0.31 | 1.14 (1.01-1.28)  0.038 | 1.09 (0.95-1.26)  0.22 | 1.02 (0.91-1.14)  0.74 |
| Model 1: Adjusted for time-updated age  Model 2: Model 1 additionally adjusted for born in Sweden, education level, baseline comorbidities (coronary heart disease, heart failure, valve disease, stroke, cancer)  Model 3: Model 2 additionally adjusted for time-updated diabetes duration  Model 3A: Model 3 additionally adjusted for time-updated mean systolic blood pressure, time-updated mean body mass index, time updated smoking status, time-updated status about blood pressure lowering medication  Model 3B: Model 3 additionally adjusted for time-updated mean high density lipoprotein, time-updated mean low density lipoprotein, time-updated status about lipid lowering mediaction  Model 3C: Model 3 additionally adjusted for time-updated insulin method  Model 3D: Model 3 additionally adjusted for time-updated albuminuria categories | | | | | | | |

### **2.8 Table S7. The impact of time-updated mean HbA1c categories on time to first atrial fibrillation using Cox regression analysis among type 2 diabetes patients – Women**

|  | **Hazard ratio (95% CI) p-value** | | | | | | |
| --- | --- | --- | --- | --- | --- | --- | --- |
| **Time to first atrial fibrillation** | **Model 1** | **Model 2** | **Model 3** | **Model 3A** | **Model 3B** | **Model 3C** | **Model 3D** |
| **Time updated mean HbA1c categories** | n events=107398 N subjects=1384149 data used = 99.7% | n events=105779 N subjects=1362613 data used = 98.1% | n events=104284 N subjects=1345102 data used = 96.9% |  |  |  |  |
| **<=6.9% (<=52 mmol/mol)** | 1.0 | 1.0 | 1.0 |  |  |  |  |
| **7.0-7.8% (53-62 mmol/mol)** | 1.06 (1.02-1.10)  0.0050 | 1.04 (1.00-1.08)  0.0270 | 1.04 (1.00-1.09)  0.0406 | 1.02 (1.00-1.06)  0.3866 | 1.03 (1.00-1.08)  0.1696 | 1.05 (1.00-1.14)  0.2723 | 1.04 (1.00-1.08)  0.1017 |
| **7.9-8.7% (63-72 mmol/mol)** | 1.16 (1.10-1.20)  <.0001 | 1.12 (1.07-1.18)  <.0001 | 1.13 (1.07- 1.20)  <.0001 | 1.08 (1.020- 1.10)  0.0080 | 1.11 (1.04-1.18)  0.0015 | 1.12 (1.02-1.22)  0.0201 | 1.11 (1.05-1.18)  0.0005 |
| **8.8-9.6% (73-82 mmol/mol)** | 1.24 (1.15-1.34)  <.0001 | 1.21 (1.12-1.31)  <.0001 | 1.18 (1.09-1.30)  <.0001 | 1.14 (1.04-1.25)  0.0036 | 1.17 (1.06-1.30)  0.0023 | 1.13 (1.00-1.30)  0.0479 | 1.17 (1.07-1.28)  0.0008 |
| **>=9.7% (>=83 mmol/mol)** | 1.27 (1.14-1.42)  <.0001 | 1.26 (1.13-1.41)  <.0001 | 1.30 (1.15-1.45)  <.0001 | 1.28 (1.13-1.46)  0.0001 | 1.37 (1.19-1.58)  <.0001 | 1.19 (1.00-1.41)  0.0474 | 1.31 (1.15-1.48)  <.0001 |
| Model 1: Adjusted for time-updated age  Model 2: Model 1 additionally adjusted for born in Sweden, education level, baseline comorbidities (coronary heart disease, heart failure, valve disease, stroke, cancer)  Model 3: Model 2 additionally adjusted for time-updated diabetes duration  Model 3A: Model 3 additionally adjusted for time-updated mean systolic blood pressure, time-updated mean body mass index, time updated smoking status, time-updated status about blood pressure lowering medication  Model 3B: Model 3 additionally adjusted for time-updated mean high density lipoprotein, time-updated mean low density lipoprotein, time-updated status about lipid lowering mediaction  Model 3C: Model 3 additionally adjusted for time-updated insulin method  Model 3D: Model 3 additionally adjusted for time-updated albuminuria categories | | | | | | | |
